# Unlocking the prognostic power of pathomics in bladder cancer: a machine learning odyssey across multiple centers

**DOI:** 10.1186/s12880-026-02353-9

**Published:** 2026-04-14

**Authors:** Jianqiu Kong, Yi Huang, Yichun Xing, Shuogui Fang, Kaiwen Tan, Juanjuan Yong, Sha Fu, Yaqiang Huang, Chun Jiang, Xinxiang Fan

**Affiliations:** 1 Department of Urology, Sun Yat-Sen Memorial Hospital, Sun Yat-Sen University, 107 Yan Jiang West Road, Guangzhou, Guangdong 510120 P. R. China; 2 Guangdong Provincial Key Laboratory of Malignant Tumor Epigenetics and Gene Regulation, Sun Yat-Sen Memorial Hospital, Sun Yat-Sen University, Guangzhou, Guangdong 510120 P. R. China; 3 Guangdong Provincial Clinical Research Center for Urological Diseases, Guangzhou, Guangdong 510120 P. R. China; 4 Department of Gynecology and Obstetrics, Sun Yat-Sen Memorial Hospital, Sun Yat-Sen University, Guangzhou, Guangdong 510120 P. R. China; 5 Department of Radiotherapy, Sun Yat-Sen Memorial Hospital, Sun Yat-Sen University, Guangzhou, Guangdong 510120 P. R. China; 6 Yunnan Key Laboratory of Artifcial Intelligence, Kunming University of Science and Technology, Kunming, 650500 China; 7 Faculty of Information Engineering and Automation, Kunming University of Science and Technology, Kunming, Yunnan 650500 P.R. China; 8 Department of Pathology, Sun Yat-Sen Memorial Hospital, Sun Yat-Sen University, Guangzhou, Guangdong 510120 P. R. China; 9 Cellular & Molecular Diagnostics Center, Sun Yat-Sen Memorial Hospital, Sun Yat-Sen University, Guangzhou, Guangdong 510120 P. R. China; 10 Department of Urology, Zhongshan City People’s Hospital, Sunwen East Road, Zhongshan, Guangdong 528400 P. R. China

**Keywords:** Bladder cancer, Pathomics, Prediction, Prognosis

## Abstract

Bladder cancer (BCa) prognostication is pivotal for tailored clinical interventions. Using machine learning, this study assesses prognostic capabilities of H&E-stained BCa images. From 569 slides across The Cancer Genome Atlas, Sun Yat-sen Memorial Hospital, and Zhongshan City People’s Hospital, we extracted 150 histopathological markers each. LASSO regression yielded a pathomic fingerprint, which was further validated. An integrated model, fusing this fingerprint with salient clinicopathological indicators, displayed notable efficacy in both training (C-index: 0.658) and validation cohorts (C-index: 0.590–0.597). Incorporating the fingerprint, age, and N stage, the model excelled in training (C-index: 0.703) and validations (C-index: 0.612–0.646). Decision curve analysis underscored its clinical relevance. Conclusively, our pathomic-clinical framework offers advanced precision in BCa patient prognosis, enhancing clinical decision-making.

**Supplementary Information:**

The online version contains supplementary material available at 10.1186/s12880-026-02353-9.

## Clinical trial number

Not applicable.

**Supplementary Information:**

The online version contains supplementary material available at 10.1186/s12880-026-02353-9.

## Introduction

Bladder cancer (BCa) is one of the most common carcinomas in the genitourinary system, ranking as the ninth most aggressive malignancy and seventh most common cancer in men [1]. BCa can be divided into two types: non-muscle-invasive bladder cancer (NMIBC) and muscle-invasive bladder cancer (MIBC). NMIBC accounts for about 3/4 of all bladder tumors and is usually treated with transurethral resection of bladder tumor (TURBt) and local chemotherapy, while MIBC often needs neoadjuvant chemotherapy and radical cystectomy (RC) [2]. MIBC is usually diagnosed de novo but may come from the 10–20% of NMIBC cases that eventually progress, which results in poor clinical outcomes. What is more, the 5-year overall survival rate of patients remains at a low level of 15%–20% [3]. Therefore, it is urgent to find out useful indicators to predict the clinical prognosis of patients with BCa.

Histopathological images contain a great deal of information about the tumor, including the nature of the lesion, degree of malignancy, and histological classification [4]. Therefore, the pathological examination is the gold standard for the diagnosis of cancer, which requires experienced pathologists to observe under the microscope [5]. However, in many parts of the world, the number of pathologists and the services they can provide may not meet the need for adequate pathological diagnosis [6]. The research and development of the digital whole slide imaging (WSI) system enables the digital reading of pathological sections, breaking the limitations of traditional microscopes.

Machine learning and deep learning are rapidly developing and have been widely used in the field of medicine in recent years [7, 8]. In particular, deep learning technology shows strong advantages in medical image processing, and is widely used in image segmentation, classification, and prediction of diseases, providing many conveniences for clinical practice [9–11]. Pathomics, as an emerging high-throughput medical image processing method, combines artificial intelligence with digital pathology and presents a blueprint for future pathological diagnosis and prognosis prediction [12, 13]. With the application of deep learning, it can obtain the characteristic changes in pathological images through artificial intelligence algorithms, which are difficult to be found by visual observation, so as to provide strong support for clinicians’ diagnosis, and improve the diagnostic accuracy and efficiency of cancer management. And the digitization in whole slide image (WSI) can provide specimen images without manual processing, which has advantages in artificial intelligence-based pathological diagnosis [14]. Recently, computational image analysis has been used to predict the diagnosis and prognosis of non-small cell lung cancer, skin cancer, kidney cancer, and breast cancer, etc. by digitizing mine features from digitized tumor histological images [15–18]. However, the application of machine learning based on digital pathology for the prognosis of BCa patients has been rarely reported.

Therefore, in this study, we will construct a prediction model by extracting the cellular morphologic features from the Hematoxylin-Eosin (H&E) stained pathological images with machine learning, for the prognosis prediction of BCa.

## Methods

### Study design and participants

In this multicentre, retrospective cohort study, we analyzed the WSIs data from 590 patients with BCa acquired from three cohorts: The Cancer Genome Atlas (TCGA, https://portal.gdc.cancer.gov), Sun Yat-sen Memorial Hospital (SYSH, Guangzhou, China), and Zhongshan City People’s Hospital (ZCPH, Zhongshan, China). BCa patients from TCGA (*N* = 367), were divided into the training cohort (*N* = 294) and the internal validation cohort (*N* = 73). We recruited 98 BCa patients treated in SYSH from October 2012 to November 2016 and 104 BCa patients treated in ZCPH from November 2009 to December 2016 as external validation cohorts 1 and 2, respectively. Retrospective evaluation of the external validation cohorts was approved by the local ethics committee and the informed consent was waived (reference SYSEC-KY-KS-2021-301 for SYMH and reference K2021-152 for ZCPH). Clinical trial number: not applicable. All patients meet the following inclusion criteria: (i) received radical cystectomy; (ii) bladder malignant tumor confirmed by pathology; (iii) complete clinicopathological data and clinical follow-up data; (iv) tumor sections with H&E staining and pathological slides with tumor content of more than 70%. All tumor samples were collected by surgical resection and were sliced at 5 μm to obtain histological sections. Baseline clinical-pathologic data, including age, sex, grade, and TNM stage were obtained from the medical records. All patients underwent standardized follow-up. Overall survival (OS) was defined as the time between surgery and death from any cause.

### Image segmentation and feature extraction

**Fig. 1:**
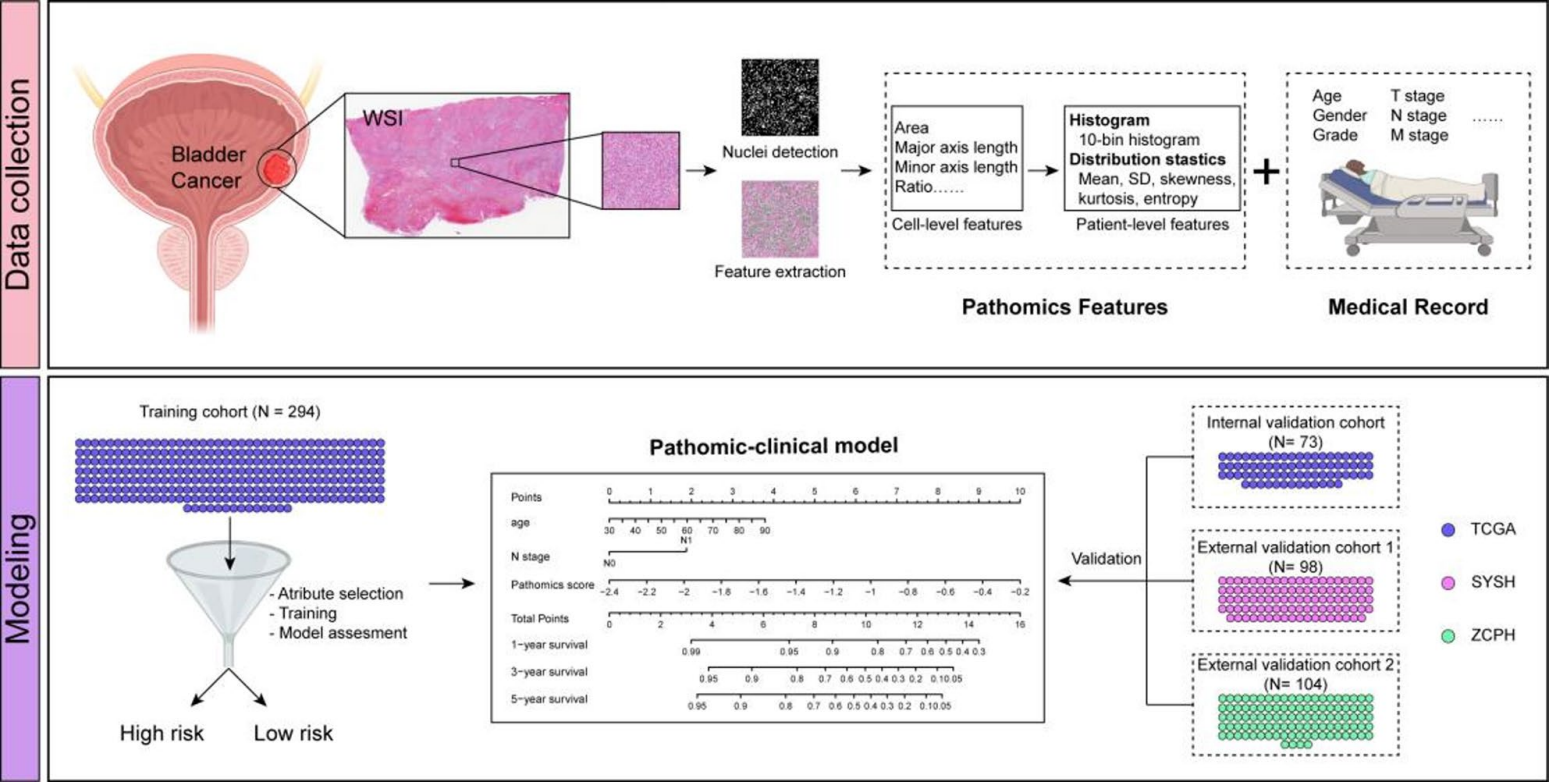
The workflow of histopathology image processing and machine learning analysis in this study. WSI, whole slide image

A flowchart outlining the analysis workflow is shown in Fig. 1. The TCGA slides were digitized at various institutions participating in the TCGA. Slides from the SYMH and ZCPH cohorts were scanned using the KF-PRO-120 scanner (KFBIO technology for health) at 40-fold magnification. To ensure comprehensive analysis, feature extraction was performed on the entire whole-slide image (WSI), not just a subsection. This approach minimizes the potential for selection bias and captures the full extent of tumor heterogeneity. The process of automated deep-learning-based histopathological image extraction, followed by image registration as previously described [19]. Matlab-R2018b (Math works, Massachusetts, USA) software was used to crop WSI and extract pathomics features. Briefly, our image feature extraction pipeline includes three steps: nucleus segmentation, cell-level feature extraction, and cell-level feature aggregation to patient-level feature (Fig. 1**)**. The stained nuclei are rich in pathological information and need to be segmented to facilitate subsequent analysis. For this, an unsupervised segmentation method that does not require parameter learning or training data through adaptive parameter setting was used. Then, 10 cell-level features were extracted from each segmented nucleus to characterize its size, shape, texture, and distance from neighboring nuclei. Finally, a 10-bin histogram (area_bin1 to area_bin10) and five distribution statistics (area_mean, area_std, area_skewness, area_kurtosis, and area_entropy) for each cell-level feature were used to aggregate the cell-level features into patient-level features. Table S1 presents the pathomics features extracted in our study.

The details of nucleus segmentation algorithm are as follows. The cell-level features are nuclear area (denoted as area), lengths of the major and minor axes of cell nucleus, and the ratio of major axis length to minor axis length (major, minor, and ratio), mean pixel values of nucleus in RGB three channels respectively (rMean, gMean, and bMean), and mean, maximum, and minimum distances (distMean, distMax, and distMin) to neighboring nuclei in the Delaunay triangulation graph. The Delaunay triangulation graph was constructed on the basis of the locations of segmented nuclei. In this graph, each nucleus was a node and connected to neighboring nuclei. After extracting 10 types of cell-level features from each segmented nucleus, we needed to aggregate the cell-level features extracted from one patient into patient-level features. For this, histogram and distribution statistics are used. To construct histogram features, a bag-of-visual-words model is used. Specifically, for each type of cell-level features, a large number of cell-level features were collected from patients and the K-means algorithm was input to learn words (i.e., clustering centers). According to sensitivity analysis, the number of clustering of k-means algorithm is fixed at 10. Cell-level features extracted from the same patient are then assigned to their nearest word using Euclidean distance, which produces a word count histogram for each patient and each cell-level feature. L1 normalization was performed on the histogram to eliminate the influence of different patient nuclei. For distribution statistics, five parameters, namely mean, standard deviation, skewness, kurtosis and entropy, are calculated for each type of cell level feature. Entropy is calculated from the normalized histogram Note that since images have different magnifications, all measurements of size and length are converted to the actual size. A total of 150 features were extracted for each patient, which was listed in Table S1.

The 150 pathomic features extracted in this study encompass several key categories, each reflecting important biological characteristics of bladder cancer. These include nuclear size and shape features, such as nuclear area, major axis length, minor axis length, and the ratio of these axes, which indicate variations in nuclear morphology and are critical indicators of cellular abnormality and malignancy. Nuclear texture features, including skewness, kurtosis, standard deviation, and entropy, quantify chromatin heterogeneity, capturing changes in chromatin organization that are associated with tumor progression and aggressiveness. Spatial relationship features, such as mean, maximum, and minimum distances between neighboring nuclei, provide insights into the spatial arrangement and organization of cells within the tumor microenvironment, which can reveal invasive growth patterns. Color and intensity features, such as mean pixel values in RGB channels, reflect variations in staining intensity that correlate with chromatin density and nuclear hyperchromasia, key indicators of tumor aggressiveness. Finally, histograms and statistical distribution features summarize the distribution of nuclear size, shape, and spatial relationships across the entire tumor, capturing tumor heterogeneity, which is a well-established factor in cancer prognosis and therapeutic resistance.

### Pathomics signature building and performance evaluation

In the training cohort, we performed the least absolute shrinkage and selection operator (LASSO) Cox regression algorithm to identify BCa-related digital pathological factors from the 150 extracted pathomics features. Then, their weighted coefficients were calculated to develop a machine learning-based prognosis model, which was afterward verified in different validation cohorts. The machine learning-based pathomics score was calculated as follows:

$$\:\mathrm{P}\mathrm{a}\mathrm{t}\mathrm{h}\mathrm{o}\mathrm{m}\mathrm{i}\mathrm{c}\mathrm{s}\:\mathrm{s}\mathrm{c}\mathrm{o}\mathrm{r}\mathrm{e}\hspace{0.17em}=\hspace{0.17em}{\mathrm{a}}_{1}{\mathrm{F}}_{1}\:+\:{\mathrm{a}}_{2}{\mathrm{F}}_{2}+\:...\:+\:{\mathrm{a}}_{\mathrm{i}}{\mathrm{F}}_{\mathrm{i}}$$

where F_i_ and a_i_ represent the selected survival-related digital pathological features and the associated regression coefficient, respectively.

The interaction of pathomics score across risk groups was tested using a likelihood ratio test and restricted cubic spline with interaction item. While the discrimination of the pathomics signature was performed by the C-index in the training cohort and validation cohorts [20, 21]. Patients were classified into high and low pathomics score subgroups based on the threshold selected by using X-tile plots.

### Prediction model development and evaluation

In the training cohort, the pathomics score and the clinicopathologic variables were included to the multivariate Cox regression algorithm. The backward stepwise selection was performed by using the Akaike’s Information Criterion (AIC) as the stopping rule to select the optimal predictors for constructing the prediction model. Then, a pathomics-clinical nomogram was constructed. C-index was measured for quantifying the discrimination of the nomogram. The calibration curve was used to assess the calibration of the nomogram. And the performance of the nomogram was validated in the validation cohorts.

### Clinical usefulness

Decision curve analysis (DCA) was performed to estimate the clinical usefulness of the nomogram by calculating the net benefits for a range of threshold probabilities. The DCA algorithm was used to evaluate alternative prognostic strategies [22].

### Statistical analysis

In this study, the X-tile software version 3.6.1 (Yale University, New Haven, CT, USA) was used to define the optimal pathomics score cut-off value, and R software version 3.6.2 (The R Foundation for Statistical Computing, https://www.r-project.org/) was used to conduct data analyses. All the R packages used in our study are detailed in the Supplementary material. All tests were two-tailed and *P* < 0.05 was regarded as significant.

## Results

### Participants

The clinicopathological characteristics of patients with BCa in the training and validation cohorts are summarized in Table 1. Of the 294 patients in the training cohort, the median age [Interquartile range (IQR)] was 68.5 (60.0–76.0) years, with 221 (75.2%) men. In the internal validation cohort, the median age (IQR) of 73 patients was 66.0 (59.0–72.0), with 52 (71.2%) men. Among the 98 patients and 104 patients in two external validation cohorts, the median ages (IQR) were 63.5 (57.3–72.0), with 88 (90.7%) men and 64.0 (57.0–70.0), with 92 (88.8%) men, respectively. In the training cohort, the 5-year overall survival (OS) was 38.3%. The 5-year OS was 35.0% in the internal validation cohort. In the external validation cohorts 1 and 2, the 5-year OS was 21.6% and 50.9%, respectively (Supplementary Material, Fig. S1).

**Table 1:** Baseline characteristics of the patients

	Training cohort (*N* = 294)	Internal validation cohort (*N* = 73)	External validation cohort 1 (*N* = 98)	External validation cohort 2 (*N* = 104)	*P* value
**Sex**					< 0.001
Male	221 (75.2%)	52 (71.2%)	88 (90.7%)	92 (88.8%)	
Female	73 (24.8%)	21 (28.8%)	10 (9.3%)	12 (11.2%)	
**Age**,** years**					< 0.001
Median (Interquartile range)	68.5 (60.0–76.0)	66.0 (59.0–76.0)	63.5 (57.3–72.0)	64.0 (57.0–70.0)	
**Grade**					< 0.001
High	278 (94.6%)	69 (94.5%)	84 (86.9%)	76 (65.5%)	
Low	16 (5.4%)	4 (5.5%)	14 (13.1%)	28 (34.5%)	
**T stage**					< 0.001
T1	9 (3.1%)	2 (2.7%)	37 (37.4%)	35 (31.0%)	
T2	135 (45.9%)	34 (46.6%)	44 (43.0%)	41 (39.6%)	
T3	119 (40.5%)	30 (41.1%)	12 (13.1%)	15 (14.7%)	
T4	31 (10.5%)	7 (9.6%)	5 (6.5%)	13 (14.7%)	
**N stage**					< 0.001
N0	170 (57.8%)	44 (60.3%)	88 (87.9%)	99 (94.0%)	
≥ N1	124 (42.2%)	29 (39.7%)	10 (12.1%)	5 (6.0%)	
**M stage**					< 0.001
M0	141 (48.0%)	32 (43.8%)	95 (96.3%)	103 (99.1%)	
M1	153 (52.0%)	41 (56.2%)	3 (3.7%)	1 (0.9%)	
**Survival status**					0.039
Alive	160 (54.4%)	41 (56.2%)	41 (41.8%)	64 (61.5%)	
Dead	134 (45.6%)	32 (43.8%)	57 (58.2%)	40 (38.5%)	
**Follow-up time**,** months**					-
Mean (range)	45.0 (0.4-165.6)	54.6 (2.3–98.5)	47.4 (1.0–78.0)	63.1 (8.9-121.6)	

Data are presented as No. (%) unless indicated otherwise

### Pathomics signature building and performance evaluation

**Fig. 2:**
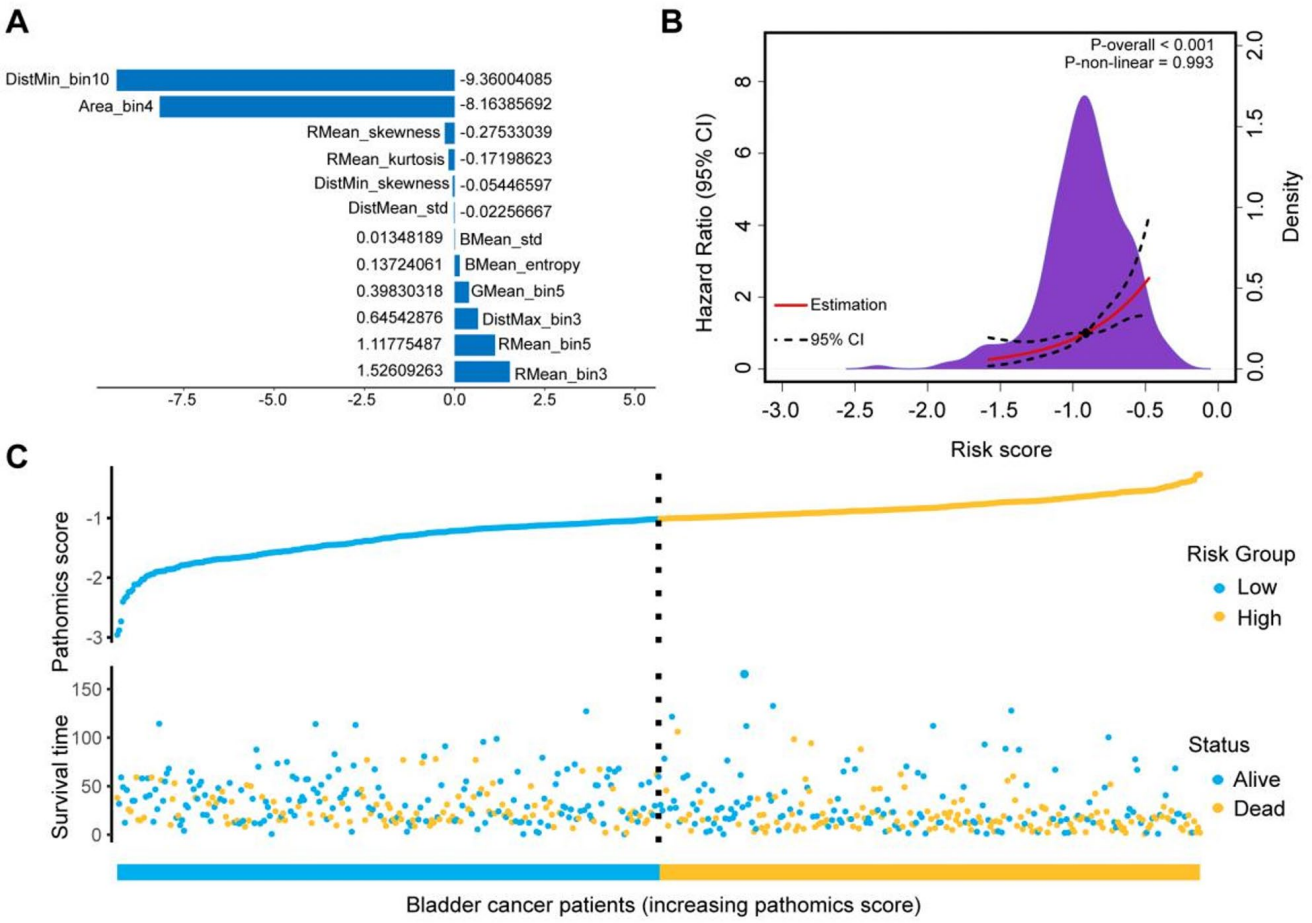
The relationship between pathomics signature and the OS in bladder cancer patients. (**A**) The histogram presents the 12 selected features and their corresponding coefficients in the pathomics signature. (**B**). Restricted cubic spline showing the relation of pathomics score with all-cause mortality. (**C**). The pathomics scores of bladder cancer patients were ranked sequentially, and patients with lower pathomics scores usually had a longer survival time compared with those with higher pathomics scores in all enrolled patients

**Fig. 3:**
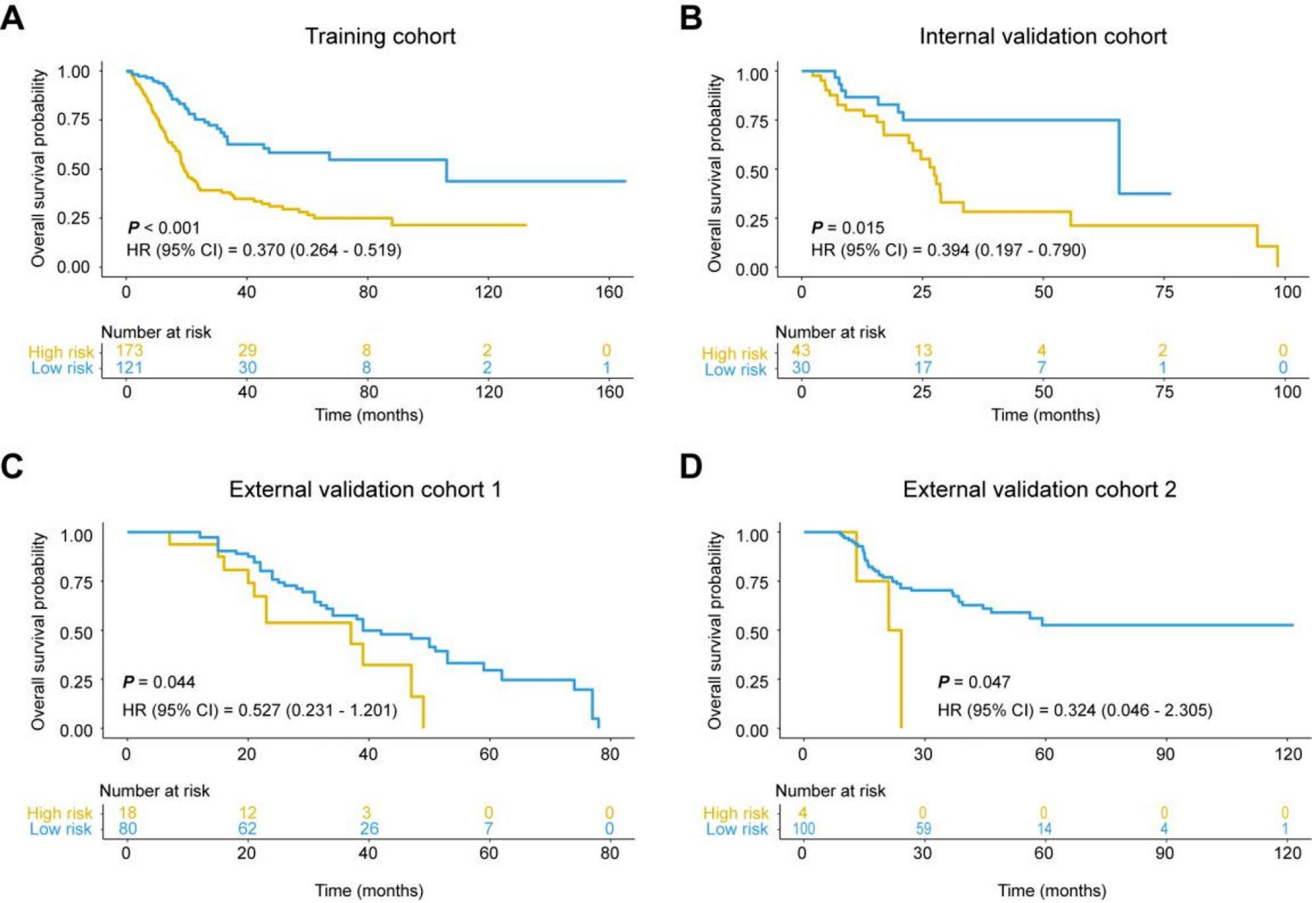
Kaplan-Meier survival analysis of the training and validation cohorts grouped by the pathomics score. OS comparison between the high and low pathomics score groups in the training cohort (**A**), internal validation cohort (**B**), external validation cohort 1 (**C**), and external validation cohort 2 (**D**). The comparisons of OS between two subgroups are performed using a two-sided log-rank test. OS, overall survival; HR, hazard ratio

Based on the training cohort, 12 specified image features (area_bin3, rMean_bin3, rMean_bin5, gMean_bin5, distMax_bin3, distMin_bin10, bMean_std, distMean_std, rMean_skewness, distMin_skewness, rMean_kurtosis, bMean_entropy) were identified from 150 pathomics features through LASSO Cox analysis with 10-fold cross-validation (Supplementary Material, Fig. S2). The identified pathomics features are shown in Fig. 2A. These selected pathomics features had only a low pairwise correlation, suggesting that they provide complementary information (Supplementary Material, Fig. S3). The pathomics signature shows a good performance in the training cohort [C-index (95%): 0.658 (0.610–0.706)], internal validation cohort [C-index (95%): 0.597 (0.480–0.714)], external validation cohort 1 [C-index (95%): 0.590 (0.511–0.669)] and external validation cohort 2 [C-index (95%): 0.596 (0.509–0.683)]. The calculation formula for the pathomics score is mentioned in the Supplementary material. Restricted cubic spline showed the relation of pathomics score with all-cause mortality (*P* for interaction < 0.001; Fig. 2B). The risk of all-cause mortality was increased with the pathomics score (*P* for non-linearity = 0.993; Fig. 2B). And all patients with high pathomics scores had lower survival rates than those with low pathomics scores (Fig. 2C). Whereafter, patients were divided into the low pathomics score group and high pathomics score group according to the optimal cutoff value (-0.96) generated by X-tile (Supplementary Material, Fig. S4). Fig. 2C presents the distributions of the pathomics score and OS status in all enrolled patients, which indicated that patients with higher pathomics scores were more likely to have death. The analysis results in each cohort are also presented in Fig. S5. The pathomics signature remained a significant prognostic indicator after stratification by clinicopathological variables, indicating the independent association of the pathomics signature with the prognosis (Supplementary Material, Fig. S6). As shown in Fig. 3, patients in the training cohort with high pathomics scores had significantly worse clinical survival outcome when compared with patients with low pathomics scores (*P* < 0.001). Notably poor prognosis in BCa patients with high pathomics scores was also demonstrated in the internal validation (*P* = 0.015), external validation 1 (*P* = 0.044) and 2 (*P* = 0.047) cohorts.

### Prediction model development and evaluation

**Fig. 4:**
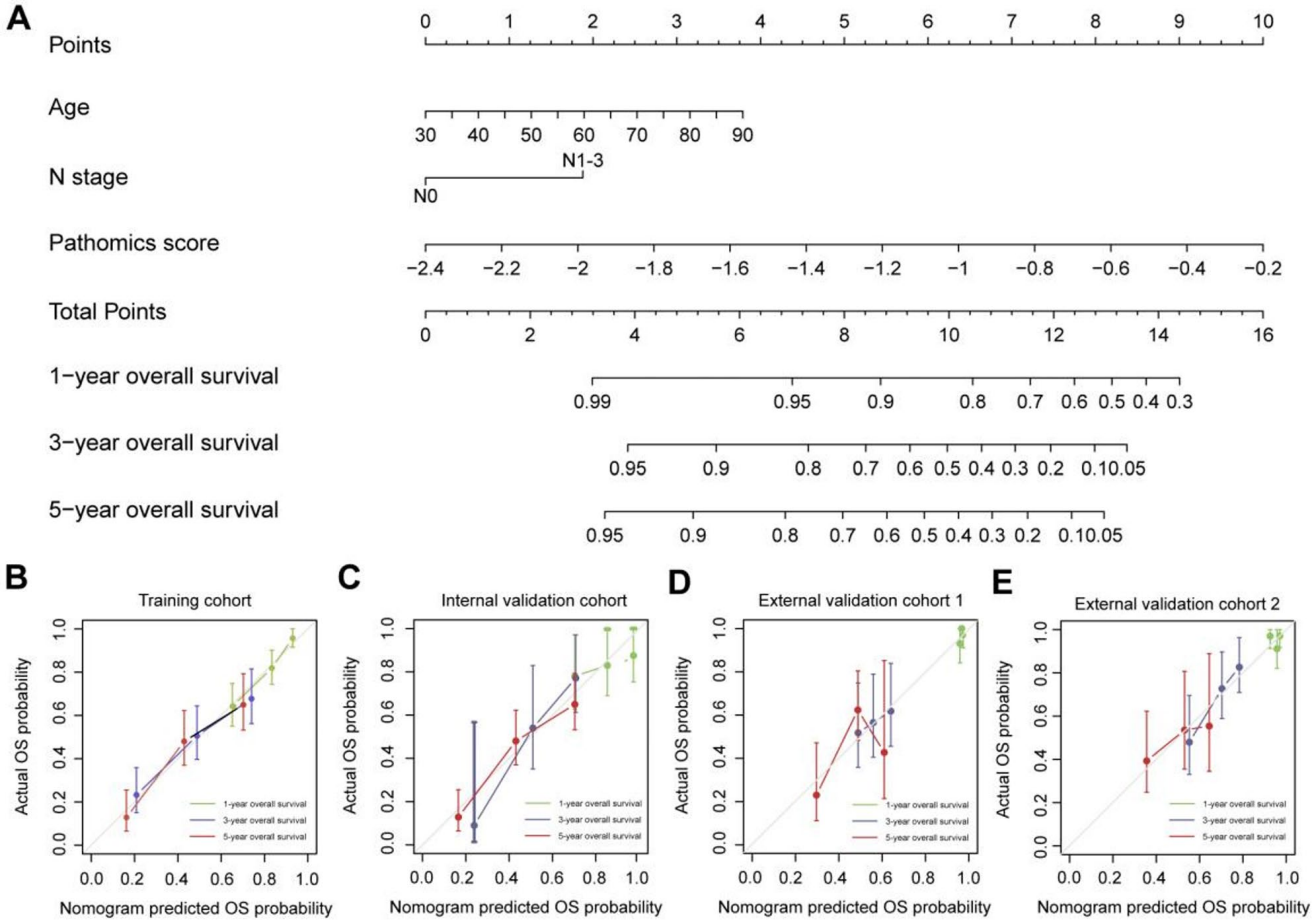
Construction and evaluation of the pathomics-clinical nomogram. (**A**) pathomics-clinical nomogram incorporating the pathomics signature, age, and N stage. (**B**-**E**). Calibration curves of the nomogram in the training cohort, internal validation, external validation cohort 1, and external validation cohort 2, respectively

Based on the multivariate Cox regression analysis, the pathomics score, age, and N stage were identified as independent predictors of OS (Table 2). The pathomics-clinical model was produced by incorporating these three independent predictors (Fig. 4A). The model yielded a C-index (95%) of 0.703 (0.656–0.750). Moreover, the calibration curves showed that 1-, 3- and 5-year OS probability predicted by the pathomics-clinical nomogram had good agreement with actual observation, indicating that its ability to predict the OS status of BCa was accurate (Fig. 4B).

**Table 2:** Cox regression analyses of the pathomics score and clinical candidate predictors in the training cohort

Variables	Univariate Cox regression		Multivariate Cox regression Pathomics-clinical model		Multivariate Cox regression Clinical model	
	HR (95% CI)	*P*	HR (95% CI)	*P*	HR (95% CI)	*P*
**The pathomics score**	8.204 (3.912 to 17.200)	< 0.001*	6.951 (3.321 to 14.547)	< 0.001*	-	-
**Sex** (male vs. female)	1.142 (0.767 to 1.700)	0.513	-	-	1.033 (1.014 to 1.052)	< 0.001*
**Age**,** years** (continuous)	1.034 (1.015 to 1.053)	< 0.001*	1.027 (1.009 to 1.046)	0.004*	-	-
**T stage**			-	-	-	-
T2	Reference	-	-	-	-	-
T1	0.700 (0.171 to 2.872)	0.620	-	-	-	-
T3	1.354 (0.937 to 1.958)	0.107	-	-	-	-
T4	1.853 (1.087 to 3.157)	0.023*	-	-	2.256 (1.595 to 3.192)	< 0.001*
**N stage** (N0 vs. ≥ N1)	2.311 (1.634 to 3.269)	< 0.001*	2.228 (1.573 to 3.155)	< 0.001*	-	-
**M stage** (M0 vs. M1)	1.253 (0.891 to 1.763)	0.196	-	-	-	-
**Grade** (high vs. low)	0.485 (0.120 to 1.972)	0.312	-	-	-	-

Abbreviations: HR, hazard ratio; CI, confidence interval

* *P* < 0.05

### Model validation and clinical application

The favorable discrimination of the pathomics-clinical model was validated in the internal validation cohort with a C-index (95%) of 0.646 (0.539–0.753). In addition, the performance was also confirmed in the external validation cohort 1 [C-index (95%CI): 0.612 (0.532–0.692)**]** and external validation cohort 2 [C-index (95%CI): 0.613 (0.522–0.704)**]**, which was higher than the clinicopathological model [Training cohort: 0.645 (0.594 to 0.696); Internal validation cohort: 0.647 (0.535 to 0.759); External validation 1:0.589 (0.516–0.662); External validation 2: 0.526 (0.453–0.599)]. Good consistency was also found in different validation cohorts (Fig. 4C-E). DCA in all four cohorts indicated that the nomogram adds more net benefit than the treat-all strategy and treat-non strategy, indicating the pathomics-clinical nomogram is clinically useful (Supplementary Material, Fig. S7). Furthermore, the ROC curves (Supplementary Material, Fig. S8) demonstrate the model’s robust discriminative ability, further supporting its clinical applicability.

## Discussion

In this study, we developed and validated an automated prognostic prediction model for BCa. By using an automated machine learning-based pathomics pipeline to identify and extract the characteristics of the nuclei in pathological images, we could identify minor changes that would not be observed by the naked eye. A pathomics signature was developed for predicting the OS of BCa patients. In addition, a pathomics-clinical model was established by incorporating the pathomics signature, age, and N stage. The pathomics-clinical model provided incremental prognostic information beyond conventional clinicopathological variables, with moderate but reproducible discrimination and favorable calibration across internal and external validation cohorts.

Currently, there are many auxiliary diagnosis methods for BCa, including cystoscopy, imaging examination, and urine cytology [23]. Pathological diagnosis is still the gold standard for the diagnosis of cancer diseases [24]. Accurate diagnosis plays a key role in subsequent treatment decisions and follow-up protocol. However, the current routine pathological diagnosis requires an experienced pathologist to observe carefully for a long time in order to obtain an accurate diagnosis. Heavy workload and strong subjectivity have become one of the main problems in pathological diagnosis [25]. In addition, minor changes in tumor lesions in the pathological sections are hard to be observed with the naked eye [26, 27]. Beyond that, the patients with the same TNM stage of BCa may have different clinical outcomes, which suggests that the current TNM staging system is not sufficient to predict prognosis,

Deep learning and machine learning are advanced technologies used in disease diagnosis and prognosis prediction in recent years [28]. Deep learning was used to build entity graph-based representations of organizations, where cell morphology and topology are embedded in each node to effectively describe the phenotypic and structural properties of the organization and can be processed through graph neural networks (GNNs) [29]. By extracting and analyzing the relevant features of images or digital pathology, the diagnosis and prognosis can be predicted [8]. Multiple studies have evaluated PD-L1/PD-1, HER-2, or other biomarkers assessments using digital scoring and AI algorithms, and have shown that digital-based techniques outperform or equal manual pathological assessments across a variety of tumor types, like NSCLC, urothelial carcinoma, melanoma, and gastric cancer [30–32]. In addition, a deep-learning based pathomics analysis of WSI images has also shown a favorable ability for predicting the prognosis of gastric cancer and colorectal cancer [33, 34]. Within this evolving field, our study extends the application of pathomics to bladder cancer prognosis using routinely available H&E whole-slide images from multiple centers.

Previous studies had proved that the combination of deep learning and pathomics features could identify subtle differences that were not easy to be observed by pathologists, which lead to different grades, stages, and prognostic results [35–37]. The features of cell nuclei play an important role in pathological image analysis, especially for the judgment of tumors, due to the fact that canceration usually begins in the nucleus. Currently, pathologists classify diseases and made a diagnosis mainly based on the cell and nuclei characteristics in pathological images, such as cell size, shape or distribution, etc. Previous studies had used the whole histopathological images or urine cytology images to make a diagnosis of BCa [15, 38]. However, there are few reports on using nuclear features in histopathological images for prognostic prediction in BCa.

H&E staining is the most common way of processing histopathological specimens, making the chromatin and nucleic acid in the nucleus violet, while the cytoplasm and extracellular matrix red. In the current study, we constructed a deep-learning-based method for automatic feature extraction of cell nuclei from H&E-stained histopathological specimens and for further prognostics prediction. In addition, we demonstrated that the pathomics features extracted from the digitized WSIs can be used to accurately predict the prognosis of BCa patients. A pathomics signature consisting of 12 pathomics features was established. Significant differences in OS between patients with high and low pathomics scores were observed in the training cohort as well as in the internal and external validation cohorts. In addition to the pathomics signature, age and N stage were identified as independent predictors of OS, which is consistent with previous studies showing that older age and nodal involvement are associated with worse survival outcomes in BCa [39, 40]. These findings suggest that quantitative nuclear morphology captured from routine H&E slides may provide clinically meaningful prognostic information beyond standard clinicopathological assessment.

Urothelial subtypes are recognized as key factors of prognosis definition after treatment with curative intent [41, 42]. From a biological perspective, the selected pathomics features may reflect several hallmarks of aggressive bladder cancer. Features related to nuclear size and axis length may capture nuclear enlargement and pleomorphism, which are classic morphological correlates of high-grade malignancy. RGB intensity-related features may reflect chromatin density, hyperchromasia, and staining heterogeneity, potentially serving as indirect surrogates of chromatin remodeling and transcriptional dysregulation. Spatial relationship features, including distances between neighboring nuclei, may reflect altered cell packing, architectural disorder, and invasive growth patterns within the tumor microenvironment. Moreover, higher-order statistical descriptors such as skewness, kurtosis, and entropy may quantify intratumoral heterogeneity in nuclear morphology and chromatin texture, which is increasingly recognized as a key determinant of tumor progression and treatment resistance.

Previous studies had reported that the quantitative image features of cancer cells can be extracted using the *CellProfiler* and showed its promising blueprints in both diagnosis and clinical outcome prediction, which indicated that the cellular morphologic features were significant in the diagnosis and prognosis prediction of cancer [43–45]. However, they developed the pathomics signature in a relatively small dataset without large external validation. In addition, they used the complex feature extraction modules and only used the typical areas of the cancer images for pathomics analysis and used a limited number of pathomics features or uninterpretable features. In the presented study, on the contrary, the cellular morphologic features can be fully automated extracted from digitized WSIs through a much simpler and more manageable algorithm. To our knowledge, this was a relatively large, multicenter, pathomics study of prognosis prediction in patients with BCa. Furthermore, the pathomics score, age, and N stage in the presented pathomics model are available from routine clinical examination. Hence, the proposed model can be used easily without any extra burden or cost to the patient.

Importantly, the present H&E-based pathomics model should not be viewed as competing with emerging biomarker platforms, but rather as a complementary and scalable component of future multimodal prognostic systems. Recent advances in bladder cancer liquid biopsy, including circulating tumor DNA, methylation-based assays, and circulating microRNAs, have demonstrated considerable promise for non-invasive monitoring, recurrence detection, and molecular risk stratification [46, 47]. Likewise, increasing evidence suggests that histological subtypes, variant histology, and molecular subtype information have important prognostic implications after curative-intent treatment [48–50]. Therefore, future computational pathology studies should aim to integrate routine histomorphology with liquid-biopsy biomarkers, subtype information, and spatially resolved microenvironmental features to achieve more biologically informed and clinically actionable prognostic stratification.

At the same time, the predictive performance of our model should be interpreted cautiously. Although statistically significant, the discrimination of both the pathomics signature and the combined pathomics-clinical model was moderate, particularly in the external validation cohorts. Therefore, our model is best regarded as an interpretable and clinically accessible prognostic framework rather than a definitive high-accuracy prediction tool. This level of performance is not uncommon in heterogeneous real-world populations, and future improvements may depend on larger datasets, more harmonized cohorts, and multimodal data integration.

Several limitations should be noted. First, this was a retrospective multicenter study, and thus potential selection bias and residual confounding cannot be excluded. Differences in clinicopathological characteristics across cohorts, as well as the imbalanced sample sizes between the training and validation sets, may also have affected model stability and generalizability. Second, to maximize clinical practicality, the present model was developed using routinely available H&E-stained WSIs; however, this design limits biological granularity, as stromal, immune, and other microenvironment-related features, as well as multimodal molecular information, were not explicitly incorporated. Third, inter-institutional variation in tissue processing, staining, scanning, and preprocessing may have influenced reproducibility. Finally, we did not perform direct benchmarking against alternative machine-learning methods. Future prospective studies with standardized workflows, larger external cohorts, and multimodal integration are warranted to further strengthen the model and support clinical translation.

## Conclusion

In conclusion, we developed and validated a pathomics-clinical model to predict the OS for patients with BCa. However, the potential biological mechanism of histopathological image features affecting survival outcomes remains to be further explored and larger prospective studies are still needed to further verify our pathomics model.

## Supplementary Information

Below is the link to the electronic supplementary material.

Supplementary Material 1

## Data Availability

The images and clinical data of the training cohort and internal validation cohort are publicly available at https://portal.gdc.cancer.gov/. Codes with annotations, comments, and instructions are available here: https://github.com/Jackeygood/BLCA-pathomics-model.git. Data from the external validation cohorts will be made available at publication upon request to the corresponding author.

## References

[1] Cumberbatch M, Jubber I, Black P, Esperto F, Figueroa J, Kamat A, Kiemeney L, Lotan Y, Pang K, Silverman D, et al. Epidemiology of Bladder Cancer: A Systematic Review and Contemporary Update of Risk Factors in 2018. Eur Urol. 2018;74(6):784–95. 30268659 10.1016/j.eururo.2018.09.001

[2] Ghandour R, Singla N, Lotan Y. Treatment Options and Outcomes in Nonmetastatic Muscle Invasive Bladder Cancer. Trends cancer. 2019;5(7):426–39. 31311657 10.1016/j.trecan.2019.05.011

[3] Cao J, Yang X, Li J, Wu H, Li P, Yao Z, Dong Z, Tian J. Screening and Identifying Immune-Related Cells and Genes in the Tumor Microenvironment of Bladder Urothelial Carcinoma: Based on TCGA Database and Bioinformatics. Front Oncol. 2019;9:1533. 32010623 10.3389/fonc.2019.01533 PMC6974676

[4] Wilson M, Fleming K. Global Cancer Care: The Role of Pathology. Am J Clin Pathol. 2016;145(1):6–7. 26712865 10.1093/ajcp/aqv030

[5] Bladder cancer. diagnosis and management of bladder cancer: © NICE (2015) Bladder cancer: diagnosis and management of bladder cancer. BJU Int. 2017;120(6):755–65. 29168333 10.1111/bju.14045

[6] Nelson A, Milner D, Rebbeck T, Iliyasu Y. Oncologic Care and Pathology Resources in Africa: Survey and Recommendations. J Clin oncology: official J Am Soc Clin Oncol. 2016;34(1):20–6. 10.1200/JCO.2015.61.9767 26578619

[7] He J, Baxter S, Xu J, Xu J, Zhou X, Zhang K. The practical implementation of artificial intelligence technologies in medicine. Nat Med. 2019;25(1):30–6. 30617336 10.1038/s41591-018-0307-0 PMC6995276

[8] Schwalbe N, Wahl B. Artificial intelligence and the future of global health. Lancet (London England). 2020;395(10236):1579–86. 32416782 10.1016/S0140-6736(20)30226-9 PMC7255280

[9] Coudray N, Ocampo PS, Sakellaropoulos T, Narula N, Snuderl M, Fenyö D, Moreira AL, Razavian N, Tsirigos A. Classification and mutation prediction from non-small cell lung cancer histopathology images using deep learning. Nat Med. 2018;24(10):1559–67. 30224757 10.1038/s41591-018-0177-5 PMC9847512

[10] McKinney SM, Sieniek M, Godbole V, Godwin J, Antropova N, Ashrafian H, Back T, Chesus M, Corrado GS, Darzi A, et al. International evaluation of an AI system for breast cancer screening. Nature. 2020;577(7788):89–94. 31894144 10.1038/s41586-019-1799-6

[11] Tschandl P, Codella N, Akay BN, Argenziano G, Braun RP, Cabo H, Gutman D, Halpern A, Helba B, Hofmann-Wellenhof R, et al. Comparison of the accuracy of human readers versus machine-learning algorithms for pigmented skin lesion classification: an open, web-based, international, diagnostic study. Lancet Oncol. 2019;20(7):938–47. 31201137 10.1016/S1470-2045(19)30333-X PMC8237239

[12] Klimov S, Miligy IM, Gertych A, Jiang Y, Toss MS, Rida P, Ellis IO, Green A, Krishnamurti U, Rakha EA, et al. A whole slide image-based machine learning approach to predict ductal carcinoma in situ (DCIS) recurrence risk. Breast Cancer Res. 2019;21(1):83. 31358020 10.1186/s13058-019-1165-5 PMC6664779

[13] Louis DN, Gerber GK, Baron JM, Bry L, Dighe AS, Getz G, Higgins JM, Kuo FC, Lane WJ, Michaelson JS, et al. Computational pathology: an emerging definition. Arch Pathol Lab Med. 2014;138(9):1133–8. 25171694 10.5858/arpa.2014-0034-ED

[14] Yu H, Gao F, Jiang L, Ma S. Development of a Whole Slide Imaging System on Smartphones and Evaluation With Frozen Section Samples. JMIR mHealth uHealth. 2017;5(9):e132. 28916508 10.2196/mhealth.8242 PMC5622289

[15] Chen S, Jiang L, Zhang E, Hu S, Wang T, Gao F, Zhang N, Wang X, Zheng J. A Novel Nomogram Based on Machine Learning-Pathomics Signature and Neutrophil to Lymphocyte Ratio for Survival Prediction of Bladder Cancer Patients. Front Oncol. 2021;11:703033. 34222026 10.3389/fonc.2021.703033 PMC8247435

[16] Ehteshami Bejnordi B, Veta M, van Johannes P, van Ginneken B, Karssemeijer N, Litjens G, van der Laak J, Hermsen M, Manson QF, Balkenhol M, et al. Diagnostic Assessment of Deep Learning Algorithms for Detection of Lymph Node Metastases in Women With Breast Cancer. JAMA. 2017;318(22):2199–210. 29234806 10.1001/jama.2017.14585 PMC5820737

[17] Esteva A, Kuprel B, Novoa RA, Ko J, Swetter SM, Blau HM, Thrun S. Dermatologist-level classification of skin cancer with deep neural networks. Nature. 2017;542(7639):115–8. 28117445 10.1038/nature21056 PMC8382232

[18] Yu KH, Zhang C, Berry GJ, Altman RB, Ré C, Rubin DL, Snyder M. Predicting non-small cell lung cancer prognosis by fully automated microscopic pathology image features. Nat Commun. 2016;7:12474. 27527408 10.1038/ncomms12474 PMC4990706

[19] Cheng J, Zhang J, Han Y, Wang X, Ye X, Meng Y, Parwani A, Han Z, Feng Q, Huang K. Integrative Analysis of Histopathological Images and Genomic Data Predicts Clear Cell Renal Cell Carcinoma Prognosis. Cancer Res. 2017;77(21):e91–100. 29092949 10.1158/0008-5472.CAN-17-0313 PMC7262576

[20] Khadhouri S, Hramyka A, Gallagher K, Light A, Ippoliti S, Edison M, Alexander C, Kulkarni M, Zimmermann E, Nathan A, et al. Machine Learning and External Validation of the IDENTIFY Risk Calculator for Patients with Haematuria Referred to Secondary Care for Suspected Urinary Tract Cancer. Eur Urol Focus. 2024;10(6):1034–42. 38906722 10.1016/j.euf.2024.06.004

[21] Khadhouri S, Gallagher KM, MacKenzie KR, Shah TT, Gao C, Moore S, Zimmermann EF, Edison E, Jefferies M, Nambiar A, et al. Developing a Diagnostic Multivariable Prediction Model for Urinary Tract Cancer in Patients Referred with Haematuria: Results from the IDENTIFY Collaborative Study. Eur Urol Focus. 2022;8(6):1673–82. 35760722 10.1016/j.euf.2022.06.001

[22] Vickers AJ, Elkin EB. Decision curve analysis: a novel method for evaluating prediction models. Med Decis Mak. 2006;26(6):565–74. 10.1177/0272989X06295361 PMC2577036 17099194

[23] Lenis A, Lec P, Chamie K, Mshs M. Bladder Cancer: A Review. JAMA. 2020;324(19):1980–91. 33201207 10.1001/jama.2020.17598

[24] Saita A, Lughezzani G, Buffi N, Hurle R, Nava L, Colombo P, Diana P, Fasulo V, Paciotti M, Elefante G, et al. Assessing the Feasibility and Accuracy of High-resolution Microultrasound Imaging for Bladder Cancer Detection and Staging. Eur Urol. 2020;77(6):727–32. 30981590 10.1016/j.eururo.2019.03.044

[25] Niazi M, Parwani A, Gurcan M. Digital pathology and artificial intelligence. Lancet Oncol. 2019;20(5):e253–61. 31044723 10.1016/S1470-2045(19)30154-8 PMC8711251

[26] Babjuk M, Böhle A, Burger M, Capoun O, Cohen D, Compérat EM, Hernández V, Kaasinen E, Palou J, Rouprêt M, et al. EAU Guidelines on Non-Muscle-invasive Urothelial Carcinoma of the Bladder: Update 2016. Eur Urol. 2017;71(3):447–61. 27324428 10.1016/j.eururo.2016.05.041

[27] Wang Y, Du L, Yang X, Li J, Li P, Zhao Y, Duan W, Chen Y, Wang Y, Mao H, et al. A nomogram combining long non-coding RNA expression profiles and clinical factors predicts survival in patients with bladder cancer. Aging. 2020;12(3):2857–79. 32047140 10.18632/aging.102782 PMC7041749

[28] Sadr H, Nazari M, Khodaverdian Z, Farzan R, Yousefzadeh-Chabok S, Ashoobi MT, Hemmati H, Hendi A, Ashraf A, Pedram MM, et al. Unveiling the potential of artificial intelligence in revolutionizing disease diagnosis and prediction: a comprehensive review of machine learning and deep learning approaches. Eur J Med Res. 2025;30(1):418. 40414894 10.1186/s40001-025-02680-7 PMC12105400

[29] Zhang S, Tong H, Xu J, Maciejewski R. Graph convolutional networks: a comprehensive review. Comput Social Networks. 2019;6(1):11. 10.1186/s40649-019-0069-y PMC10615927 37915858

[30] Baxi V, Lee G, Duan C, Pandya D, Cohen DN, Edwards R, Chang H, Li J, Elliott H, Pokkalla H, et al. Association of artificial intelligence-powered and manual quantification of programmed death-ligand 1 (PD-L1) expression with outcomes in patients treated with nivolumab ± ipilimumab. Mod Pathol. 2022;35(11):1529–39. 35840720 10.1038/s41379-022-01119-2 PMC9596372

[31] Wu J, Liu C, Liu X, Sun W, Li L, Gao N, Zhang Y, Yang X, Zhang J, Wang H, et al. Artificial intelligence-assisted system for precision diagnosis of PD-L1 expression in non-small cell lung cancer. Mod Pathol. 2022;35(3):403–11. 34518630 10.1038/s41379-021-00904-9

[32] Zeng Q, Klein C, Caruso S, Maille P, Laleh NG, Sommacale D, Laurent A, Amaddeo G, Gentien D, Rapinat A, et al. Artificial intelligence predicts immune and inflammatory gene signatures directly from hepatocellular carcinoma histology. J Hepatol. 2022;77(1):116–27. 35143898 10.1016/j.jhep.2022.01.018

[33] Chen D, Fu M, Chi L, Lin L, Cheng J, Xue W, Long C, Jiang W, Dong X, Sui J, et al. Prognostic and predictive value of a pathomics signature in gastric cancer. Nat Commun. 2022;13(1):6903. 36371443 10.1038/s41467-022-34703-w PMC9653436

[34] Wang R, Dai W, Gong J, Huang M, Hu T, Li H, Lin K, Tan C, Hu H, Tong T, et al. Development of a novel combined nomogram model integrating deep learning-pathomics, radiomics and immunoscore to predict postoperative outcome of colorectal cancer lung metastasis patients. J Hematol Oncol. 2022;15(1):11. 35073937 10.1186/s13045-022-01225-3 PMC8785554

[35] Chandramouli S, Leo P, Lee G, Elliott R, Davis C, Zhu G, Fu P, Epstein JI, Veltri R, Madabhushi A. Computer extracted features from initial h&e tissue biopsies predict disease progression for prostate cancer patients on active surveillance. Cancers (Basel). 2020;12(9). 10.3390/cancers12092708 PMC7563653 32967377

[36] Cheng J, Han Z, Mehra R, Shao W, Cheng M, Feng Q, Ni D, Huang K, Cheng L, Zhang J. Computational analysis of pathological images enables a better diagnosis of TFE3 Xp11.2 translocation renal cell carcinoma. Nat Commun. 2020;11(1):1778. 32286325 10.1038/s41467-020-15671-5 PMC7156652

[37] Xu H, Park S, Hwang TH. Computerized Classification of Prostate Cancer Gleason Scores from Whole Slide Images. IEEE/ACM Trans Comput Biol Bioinform. 2020;17(6):1871–82. 31536012 10.1109/TCBB.2019.2941195

[38] Awan R, Benes K, Azam A, Song TH, Shaban M, Verrill C, Tsang YW, Snead D, Minhas F, Rajpoot N. Deep learning based digital cell profiles for risk stratification of urine cytology images. Cytometry A. 2021;99(7):732–42. 33486882 10.1002/cyto.a.24313

[39] Masson-Lecomte A, Vordos D, Hoznek A, Yiou R, Allory Y, Abbou CC, de la Taille A, Salomon L. External validation of extranodal extension and lymph node density as predictors of survival in node-positive bladder cancer after radical cystectomy. Ann Surg Oncol. 2013;20(4):1389–94. 23208127 10.1245/s10434-012-2753-0

[40] Oddens JR, Sylvester RJ, Brausi MA, Kirkels WJ, van de Beek C, van Andel G, de Reijke TM, Prescott S, Witjes JA, Oosterlinck W. The effect of age on the efficacy of maintenance bacillus Calmette-Guérin relative to maintenance epirubicin in patients with stage Ta T1 urothelial bladder cancer: results from EORTC genito-urinary group study 30911. Eur Urol. 2014;66(4):694–701. 24948466 10.1016/j.eururo.2014.05.033

[41] Claps F, Biasatti A, Di Gianfrancesco L, Ongaro L, Giannarini G, Pavan N, Amodeo A, Simonato A, Crestani A, Cimadamore A et al. The prognostic significance of histological subtypes in patients with muscle-invasive bladder cancer: an overview of the current literature. J Clin Med. 2024;13(15). 10.3390/jcm13154349 PMC11313590 39124615

[42] Basile G, de Angelis M, Leni R, Re C, Longoni M, Mari A, Soria F, Pradere B, Del Giudice F, Laukhtina E, et al. Implications for diagnosis and treatment strategies in non-muscle invasive bladder cancer with variant histology: a systematic review. Minerva Urol Nephrol. 2023;75(3):278–88. 36946716 10.23736/S2724-6051.23.05091-7

[43] Mejia I, Chen YC, Díaz B. Analysis of Golgi Morphology Using Immunofluorescence and CellProfiler Software. Methods Mol Biol. 2023;2557:765–84. 36512250 10.1007/978-1-0716-2639-9_46

[44] Schüssele DS, Haller PK, Haas ML, Hunter C, Sporbeck K, Proikas-Cezanne T. Autophagy profiling in single cells with open source CellProfiler-based image analysis. Autophagy. 2023;19(1):338–51. 35435815 10.1080/15548627.2022.2065617 PMC9809960

[45] Stirling DR, Carpenter AE, Cimini BA. CellProfiler Analyst 3.0: accessible data exploration and machine learning for image analysis. Bioinformatics. 2021;37(21):3992–4. 34478488 10.1093/bioinformatics/btab634 PMC10186093

[46] Crocetto F, Amicuzi U, Musone M, Magliocchetti M, Di Lieto D, Tammaro S, Pastore AL, Fuschi A, Falabella R, Ferro M, et al. Liquid Biopsy: current advancements in clinical practice for bladder cancer. J Liquid Biopsy. 2025;9. 10.1016/j.jlb.2025.100310 PMC12281373 40698358

[47] Cicatiello AG, Musone M, Imperatore S, Giulioni C, La Rocca R, Cafarelli A, Del Giudice F, Dentice M, Crocetto F. Circulating miRNAs in genitourinary cancer: pioneering advances in early detection and diagnosis. J Liquid Biopsy. 2025;8. 10.1016/j.jlb.2025.100296 PMC12088820 40391154

[48] Black AJ, Black PC. Variant histology in bladder cancer: diagnostic and clinical implications. Transl Cancer Res. 2020;9(10):6565–75. 35117266 10.21037/tcr-20-2169 PMC8798576

[49] Takahara T, Murase Y, Tsuzuki T. Urothelial carcinoma: variant histology, molecular subtyping, and immunophenotyping significant for treatment outcomes. Pathology. 2021;53(1):56–66. 33070956 10.1016/j.pathol.2020.09.004

[50] Ornaghi PI, Tafuri A, Orlando R, Panunzio A, Moschini M, Afferi L, Lonati C, Cerruto MA, Antonelli A. Surgical and functional outcomes after robot-assisted radical cystectomy in female patients: a systematic review of the literature. Mini-invasive Surg. 2021;5:42.

