# Supplementary material for: Unlocking the prognostic power of pathomics in bladder cancer: a machine learning odyssey across multiple centers
Source: BMC Med Imaging. 2026 Apr 14;26:266. doi: 10.1186/s12880-026-02353-9 (PMC13366761; doi:10.1186/s12880-026-02353-9)
Supplement: Supplementary file 1 — Supplementary Material 1 [file 12880_2026_2353_MOESM1_ESM.docx]

Supporting information for

**Unlocking the Prognostic Power of Pathomics in Bladder Cancer: A Deep Learning Odyssey Across Multiple Centers**

**Authors:** Jianqiu Kong^1,2,3†^, Yi Huang^1,2,3†^, Yichun Xing^4†^, Shuogui Fang^5†^, Kaiwen Tan^6, 7^, Juanjuan Yong^8^, Sha Fu^8, 9^, Yaqiang Huang^10*^, Chun Jiang^1,2,3*^, Xinxiang Fan^1,2,3*^

**Methods**

***Aggregation of cell-level features into patient-level features***

The details of nucleus segmentation algorithm are as follows. The cell-level features are nuclear area (denoted as area), lengths of the major and minor axes of cell nucleus, and the ratio of major axis length to minor axis length (major, minor, and ratio), mean pixel values of nucleus in RGB three channels respectively (rMean, gMean, and bMean), and mean, maximum, and minimum distances (distMean, distMax, and distMin) to neighboring nuclei in the Delaunay triangulation graph. The Delaunay triangulation graph was constructed on the basis of the locations of segmented nuclei. In this graph, each nucleus was a node and connected to neighboring nuclei. After extracting 10 types of cell-level features from each segmented nucleus, we needed to aggregate the cell-level features extracted from one patient into patient-level features. For this, histogram and distribution statistics are used. To construct histogram features, a bag-of-visual-words model is used. Specifically, for each type of cell-level features, a large number of cell-level features were collected from patients and the K-means algorithm was input to learn words (i.e., clustering centers). According to sensitivity analysis, the number of clustering of k-means algorithm is fixed at 10. Cell-level features extracted from the same patient are then assigned to their nearest word using Euclidean distance, which produces a word count histogram for each patient and each cell-level feature. L1 normalization was performed on the histogram to eliminate the influence of different patient nuclei. For distribution statistics, five parameters, namely mean, standard deviation, skewness, kurtosis and entropy, are calculated for each type of cell level feature. Entropy is calculated from the normalized histogram Note that since images have different magnifications, all measurements of size and length are converted to the actual size.

***Detailed description of the LASSO method***

LASSO is a powerful method for regression with high dimensional predictors. In our study, the LASSO method was combined with Cox regression model for analysis of the prognosis of BCa patients, which could select the most important predictive features from the training cohort. This method minimizes a log partial likelihood subject to the sum of the absolute values of the parameters being bounded by a constant:

$\hat{\text{β}}$= argmin ℓ(β), subject to ⅀|β_j_| ≤ s

where, $\hat{\text{β}}$is the obtained parameters, ℓ(β) is the log partial likelihood of the Cox regression model, s＞0 is a constant. The LASSO method can be used for feature reduction and selection by shrinking coefficients and forcing certain coefficients to be set to zero through absolute constraint. In this study, the standardized constraint parameter s was set as 0.03229 and 12 nonzero coefficients ($\hat{\text{β}}$) were selected by LASSO.

***R packages used in this study***

All statistical analyses were performed using R statistical software version 3.6.2 (https://www.r-project.org/). R packages used in this study are listed as follow:

| **Statistical analysis** | **R package** | **Version** |
| --- | --- | --- |
| LASSO Cox regression | glmnet | 4.1-3 |
| Cox regression, nomogram construction, calibration curve | rms | 6.2-0 |
| Heatmap | pheatmap | 1.0.12 |
| Restricted cubic spline | rms | 6.2-0 |
| Decision curve analysis | dca.R | 1.1 |
| Kaplan-Meier survival analysis | ggsurvplot | 0.4.9 |

***Calculation formula for the pathomics score***

Pathomic score = - 8.16385692 * area_bin4

+ 1.52609263 * rMean_bin3

+ 1.11775487 * rMean_bin5

+ 0.39830318 * gMean_bin5

+ 0.64542876 * distMax_bin3

- 9.36004085 * distMin_bin10

+ 0.01348189 * bMean_std

- 0.02256667 * distMean_std

- 0.27533039 * rMean_skewness

- 0.05446597 * distMin_skewness

- 0.17198623 * rMean_kurtosis

+ 0.13724061 * bMean_entropy

**Figures**

**Supplementary Figure 1**. Kaplan-Meier survival analysis of the training and validation cohorts. (A) The OS curve in the training cohort. (B) The OS curve in the internal validation cohort. (C) The OS curve in the external validation cohort 1. (D) The OS curve in the external validation cohort 2. *Abbreviations*: OS, overall survival.


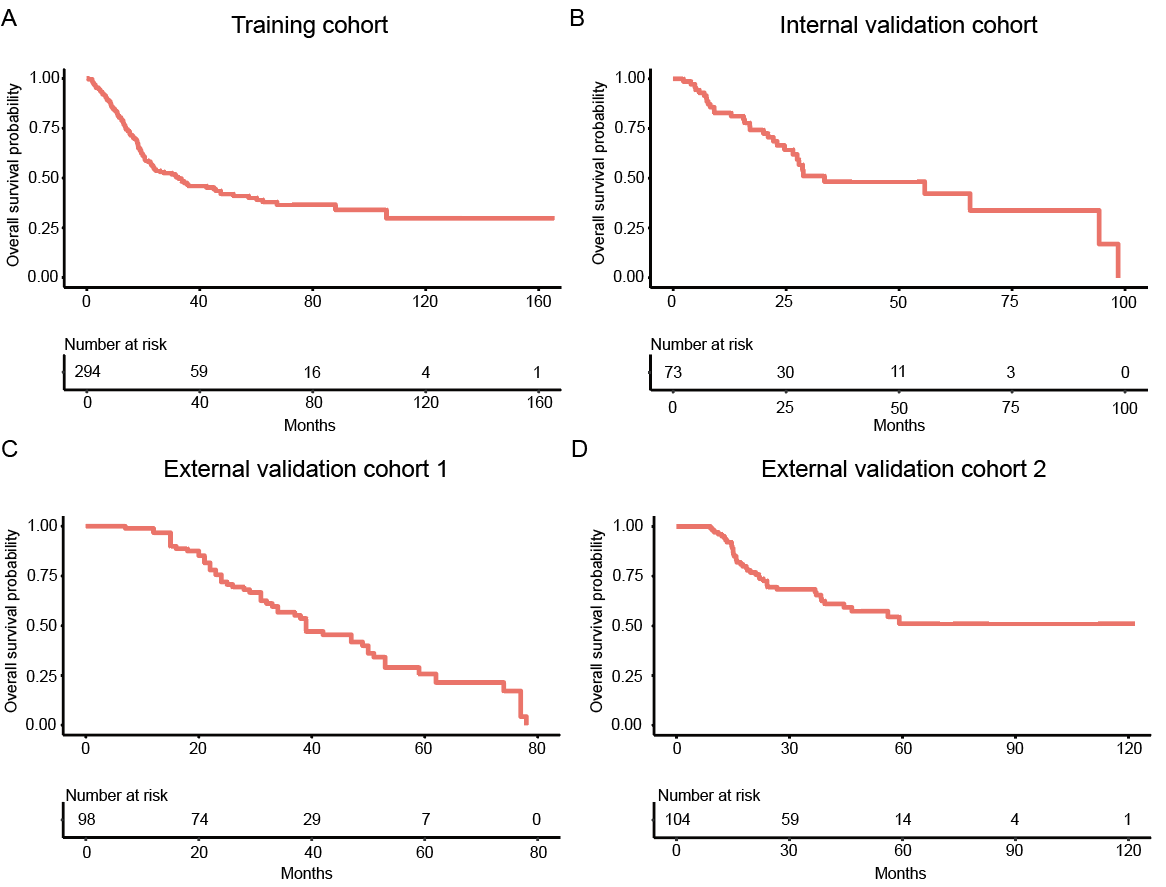


**Supplementary Figure 2.** Pathomics features selection using the LASSO regression algorithm. (A) Selection of the tuning parameter (λ). The tuning parameter lambda (λ) was selected by the LASSO method based on 10-fold cross-validation via minimum criteria. The binomial deviance was plotted versus the log-transformed λ. Based on the minimum criteria, the calculated optimal values were plotted as the dotted vertical line. The optimal λ value of 0.03229 with log (λ) of –3.433 was selected. (B) LASSO coefficient profiles of the 150 nucleus features. Twelve stable features with nonzero coefficients were selected, according to the vertical line plotted at the optimal λ value. *Abbreviations:* LASSO, least absolute shrinkage and selection operator.


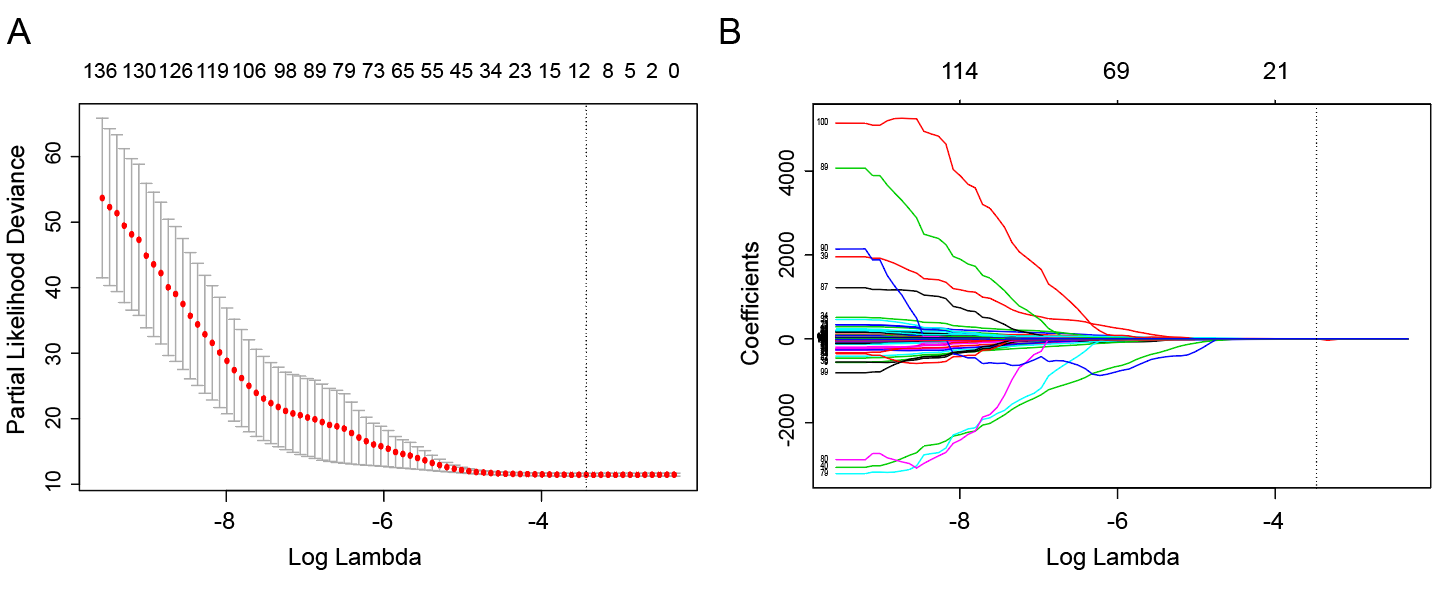


**Supplementary Figure 3**. Correlation analysis between the selected features. The Pearson correlation coefficients were calculated, and values closer to 1 identify a better correlation.


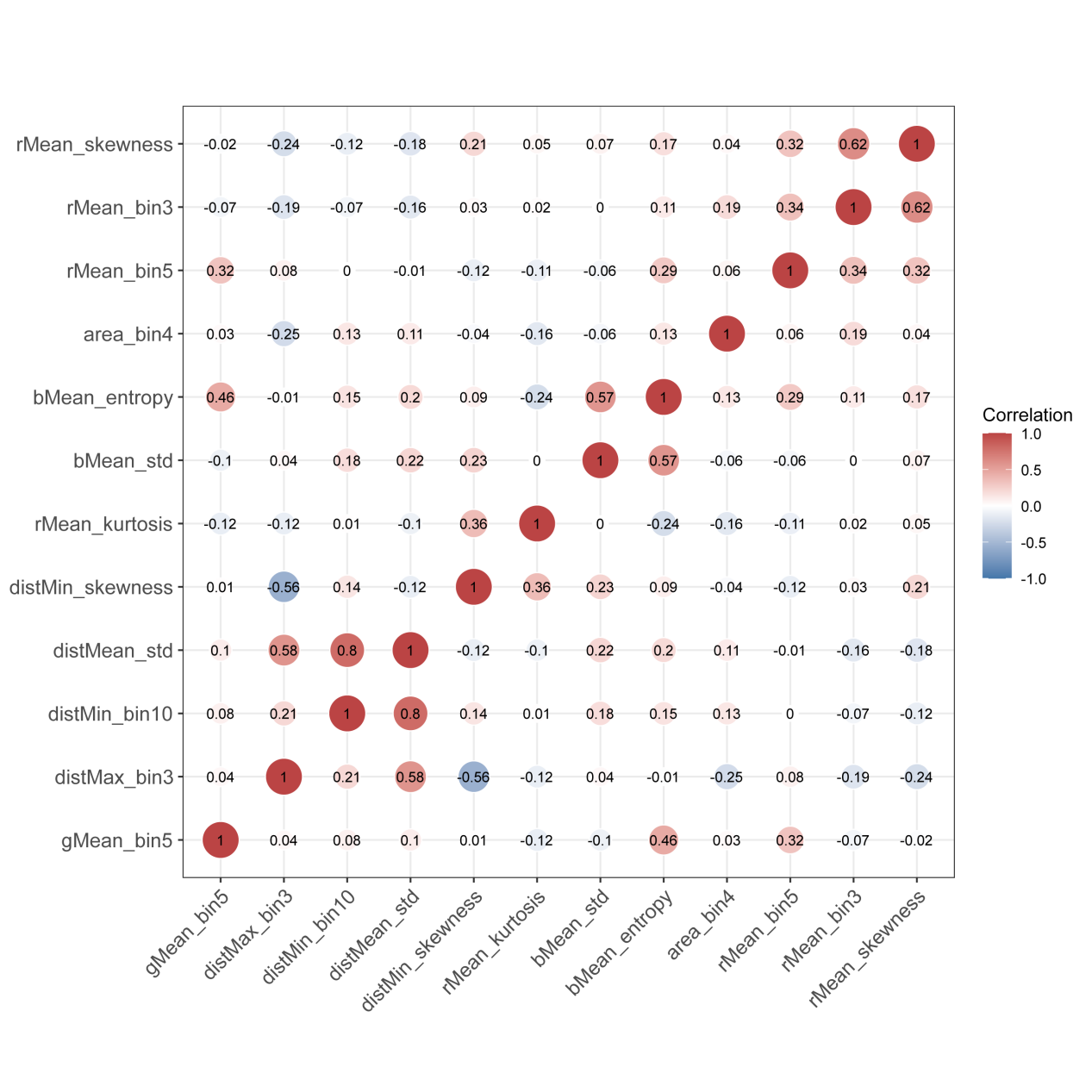


**Supplementary Figure 4**. X-tile plots identifying the optimal pathomics score cutoff value. (A) The colors in the plot represent the strength of the association at each division, ranging from low (black) to high (bright red or green). Red represents the inverse association between the pathomics signature and survival, whereas green represents a positive association. The x-axis represents all potential cutoff points, from low to high (left to right), that define a low subset, whereas the y-axis represents cutoff points from high to low (top to bottom) that define a high subset. The optimum cutoff point is highlighted by the black dot on the x-axis. (B) The cutoff value of the pathomics signature is -0.96 and the numbers of patients in high score subgroup is 173 while in low score subgroup is 121.


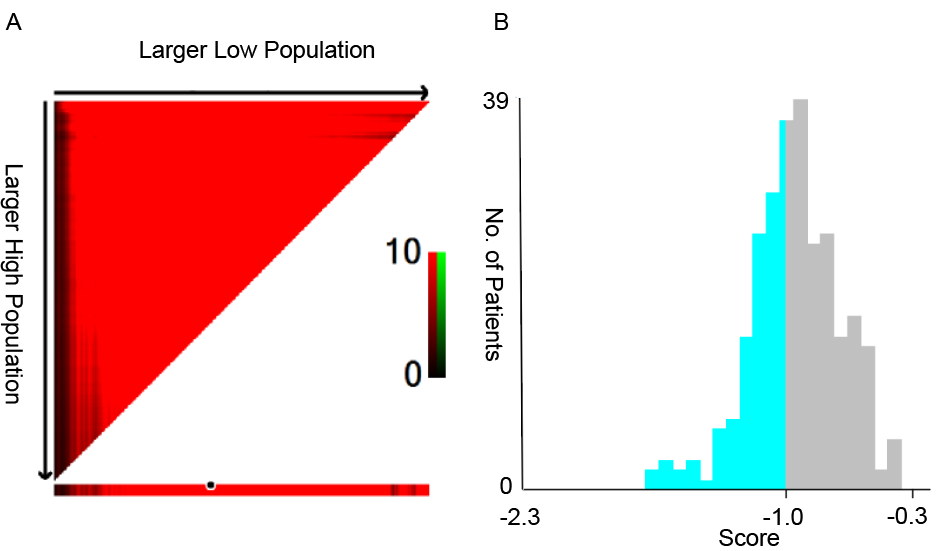


**Supplementary Figure 5**. Distribution of pathomics score according to the survival status and time in the training cohort (A), internal validation cohort (B), external validation cohort 1 (C) and external validation cohort 2 (D).


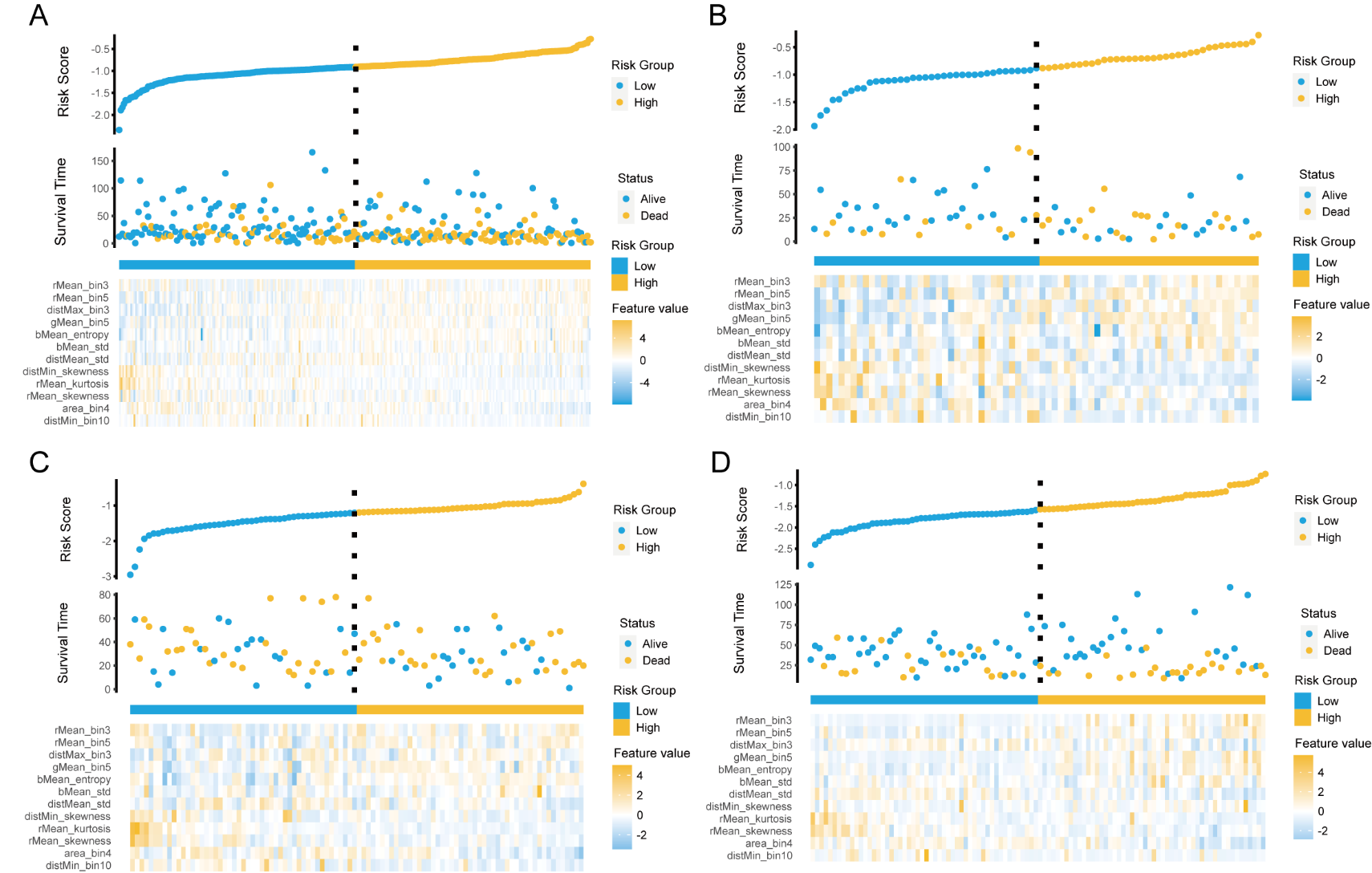


**Supplementary Figure 6**. Kaplan-Meier survival analysis of the OS of all 569 patients according to the pathomics score level stratified by various clinicopathological variables. The comparisons of OS between two groups are performed using a two-sided log-rank test.


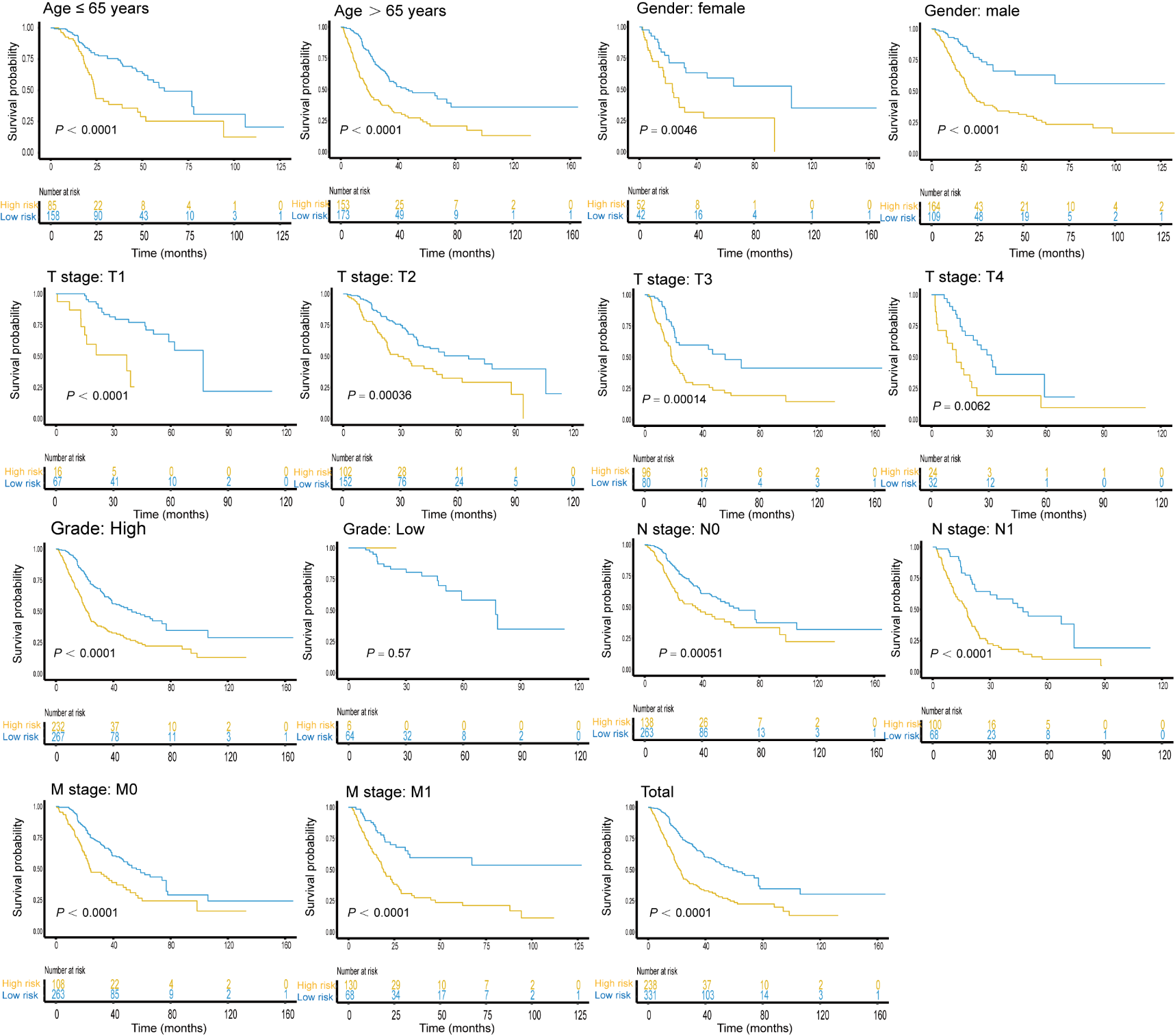


**Supplementary Figure 7.** Decision curve analysis for the nomogram. The color line represents the competing-risk nomogram (light green presents the 1-year OS, purple line presents the 3-year OS, and red line represents the 5-year OS), the gray line represents the treat-all scheme, and the black line represents the treat-none scheme.


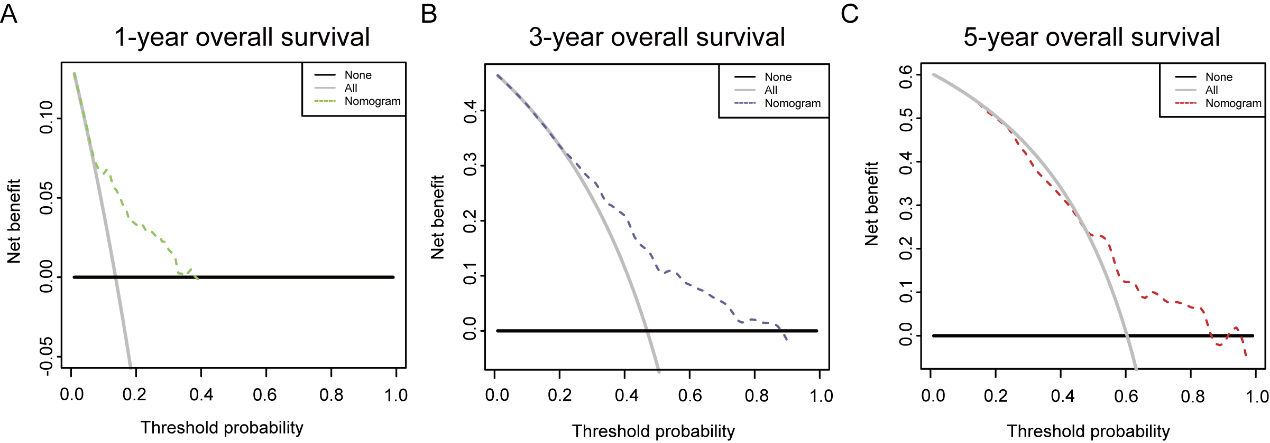


**Supplementary Figure 8.** ROC curves for the combined pathomics-clinical model showing 1-year, 3-year, and 5-year survival predictions across the training cohort, internal validation cohort, and two external validation cohorts. AUC values are provided for each time point to evaluate the model’s performance in predicting survival outcomes.


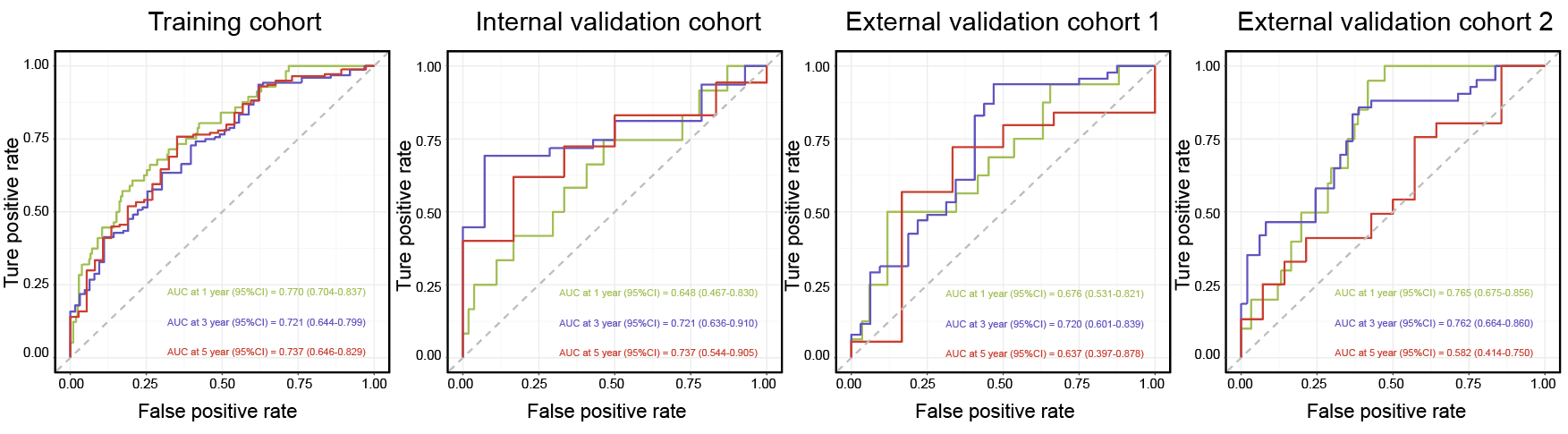


**Table**

**Supplementary Table 1**. Comprehensive list of quantitative pathomics features extracted in our study.

| Feature classification | Features description | Feature number |
| --- | --- | --- |
| Histogram | Area_binα  Major_binα  Minor_binα  Ratio_binα  rMean_binα  gMean_binα  bMean_binα  distMean_binα  distMax_binα  distMIn_binα | 100 |
| Distribution statistics | β_mean  β_std  β_skewness  β_kurtosis  β_entropy | 50 |

α represents the 10-bin histogram, which could be 1, 2, 3, 4, …, 9, 10;

β represents the type of cell-level features, which could be area, major, minor, ratio, rMean, gMean, bMean, distMean, distMax and distMin.
